# Measuring the impact of health policies using Internet search patterns: the case of abortion

**DOI:** 10.1186/1471-2458-10-514

**Published:** 2010-08-25

**Authors:** Ben Y Reis, John S Brownstein

**Affiliations:** 1 Children's Hospital Informatics Program, Harvard-MIT Division of Health Sciences and Technology, Children's Hospital Boston, Harvard Medical School, Boston, MA, USA

## Abstract

**Background:**

Internet search patterns have emerged as a novel data source for monitoring infectious disease trends. We propose that these data can also be used more broadly to study the impact of health policies across different regions in a more efficient and timely manner.

**Methods:**

As a test use case, we studied the relationships between abortion-related search volume, local abortion rates, and local abortion policies available for study.

**Results:**

Our initial integrative analysis found that, both in the US and internationally, the volume of Internet searches for abortion is inversely proportional to local abortion rates and directly proportional to local restrictions on abortion.

**Conclusion:**

These findings are consistent with published evidence that local restrictions on abortion lead individuals to seek abortion services outside of their area. Further validation of these methods has the potential to produce a timely, complementary data source for studying the effects of health policies.

## Background

Traditional health surveillance techniques are limited in their abilities to measure the impact of health policies in a timely and geographically comprehensive fashion. Emerging surveillance approaches that track patterns of online searches have the potential to provide timely global population health monitoring across a range of health indicators [1]. These search data are currently being used to detect and monitor emerging infectious diseases, including influenza [2-5]. We propose that these data can also be used more broadly to study the impact of public health policies at fine geographic scales and with fewer delays in data collection.

As an example of the potential value of search query data, we focus on the relationship between local abortion policies and local abortion rates, both in the US and internationally. Abortion policies and their effects have been widely researched, and their impact continues to be intensely debated around the world. Major issues include differences in ethical and cultural approaches towards abortion and concerns about patient safety [6-10]. The potential to measure the relationships between local policy decisions and local abortion rates in a more efficient manner could have significant implications for public health planning, monitoring and intervention.

We propose that Internet search patterns could provide a complementary information source for understanding the impact of abortion policies in a specific region. When analyzed in conjunction with information on the region's abortion policies, these data could be used to study the relationships between abortion policies and rates. In this brief report, we describe an initial proof-of-concept evaluation of these methods.

## Methods

For our initial analysis, we examined four types of abortion data in each of the 50 US states: 1. Abortion rates - percentage of all pregnancies that end in abortions, excluding fetal deaths and miscarriages; 2. Abortion policies, such as mandatory waiting periods and parental consent; 3. Abortion availability - percentage of counties with providers; and 4. Legal context, including current and past legislation. We compared these data to abortion search volume by state, defined as relative Internet search volume for the term "abortion", normalized by total Internet search volume for that state. We also compared abortion search volume and abortion rates and restrictions across 37 different countries, using 19 search terms to cover the term "abortion" across the major languages used in these countries. All search statistics examined were anonymous summary statistics and were further aggregated over an entire state or country over an entire year in order to address potential privacy concerns surrounding the analysis of Internet search data.

A comprehensive set of abortion and search data were available for 2004. Abortion statistics were obtained from the United Nations (data freely available at http://unstats.un.org), the United States Centers for Disease Control and Prevention (cdc.gov), the Gutmacher institute http://www.guttmacher.org, and the Johnston Archive http://www.johnstonsarchive.net. Data on search volume were obtained from Google Insights for Search http://www.google.com/insights/search/, representing the proportion of Google Web searches performed in a particular geographic region relative to the total number of searches performed in that region. Regions with insufficient data were excluded. We used the following 19 search terms to cover abortion in multiple languages: abortion, aborto, abortus, avortement, аборт, 流 产, 中 絶 pobačaj, potrat, abort (Scandinavia only), abortti, abtreibung, abortusz, הלפה, abortas, aborcji, aborcja, aborcje, and avort. Search volumes for the different search terms were scaled against common reference statistics to allow for comparison across languages. Data regarding restrictions on abortion for each country included whether abortion was available on request, for economic or social reasons, to preserve mental health, to preserve physical health, in cases of fetal impairment, in cases of rape or incest, or to save a woman's life http://unstats.un.org. All data regarding abortions referred to legal abortions that were reported, and do not account for the potential impact of illegal abortions that most often go unreported.

## Results

**Figure 1:**
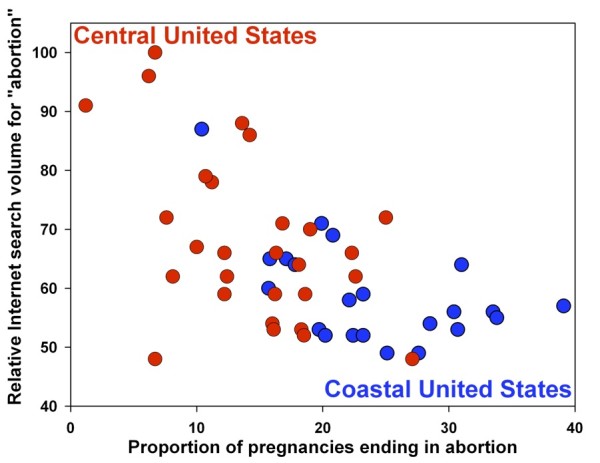
**Relationship between abortion search volume and abortion rates in the United States**. Data for the 50 U.S. States in 2004 shows a strong *inverse *correlation between abortion search volume and local abortion rates (Spearman's Rho -0.547, p < 0.001). A strong direct relationship is seen between restrictions and abortion search volume (p = 0.005).

**Figure 2:**
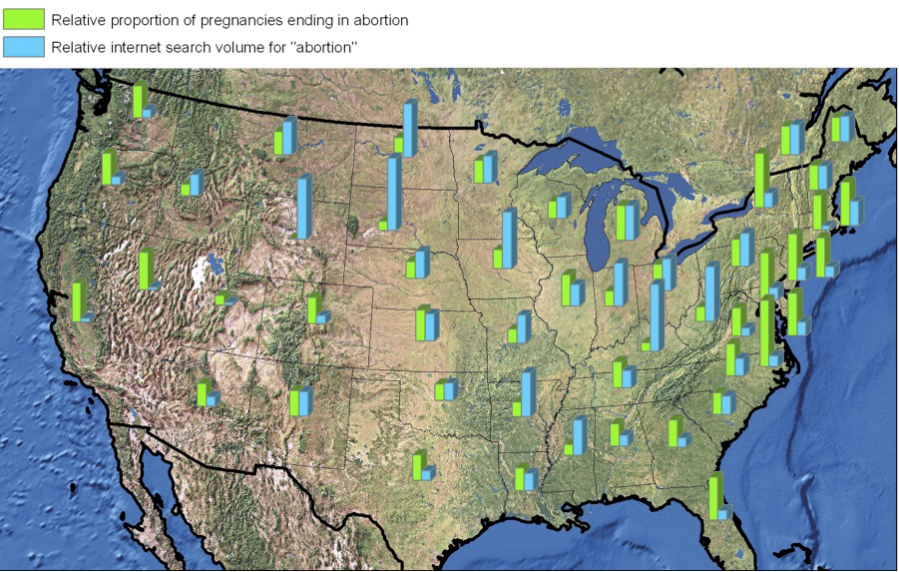
**Geographic visualization comparing abortion search volume and abortion rates by US state**.

Looking at abortion rates across the 50 US states, abortion search volume had a strong *inverse *correlation to local abortion rates (Spearman's Rho -0.547, p < 0.001.; Figures 1, 2. With regard to the abortion policies available for study, abortion search volume was significantly higher in states having any of the following four restrictions: mandatory waiting period, mandatory counseling, mandatory parental notification in the case of minors, and mandatory parental consent for minors (sample means: 66.53 vs. 57.71; 95% C.I. for true mean difference: 2.77 - 14.85; p-value of two-sample two-tailed t-test: 0.005). Examining abortion availability, abortion search volume was significantly higher in states where fewer than 10% of counties have providers (means: 69.76 vs. 58.36; 95% C.I.: 4.78 - 18.02; p = 0.001). Analyzing the effects of legal context, laws stipulating that late-term abortions would become illegal if Roe vs. Wade were repealed were associated with higher search volumes (means: 67.68 vs. 59.45; 95% C.I.: 1.47 - 14.98; p = 0.018), while laws indicating that abortion would remain legal were associated with lower search volumes (means: 54.43 vs. 65.63; 95% C.I.: -5.00 to -17.40; p = 0.001). Historic pre-Roe abortion bans were also associated with higher search volume (means: 68.13 vs. 57.42; 95% C.I: 4.61-16.81; p = 0.001).

**Figure 3:**
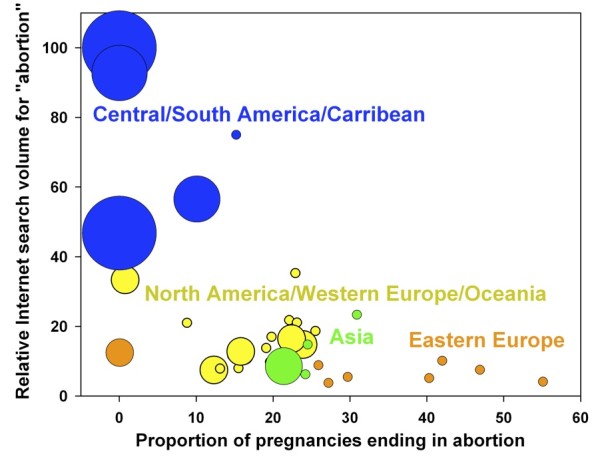
**Relationship between abortion search volume and abortion rates across 37 countries**. Data from 37 countries also reveals an inverse correlation between abortion search volume and local abortion rates (Rho = -0.484, p = 0.004), with certain geographic regions showing general trends. Marker size indicates the number of restrictions (from 0 to 7) that exist on abortion in each country according to UNStats (See SOM).

**Figure 4:**
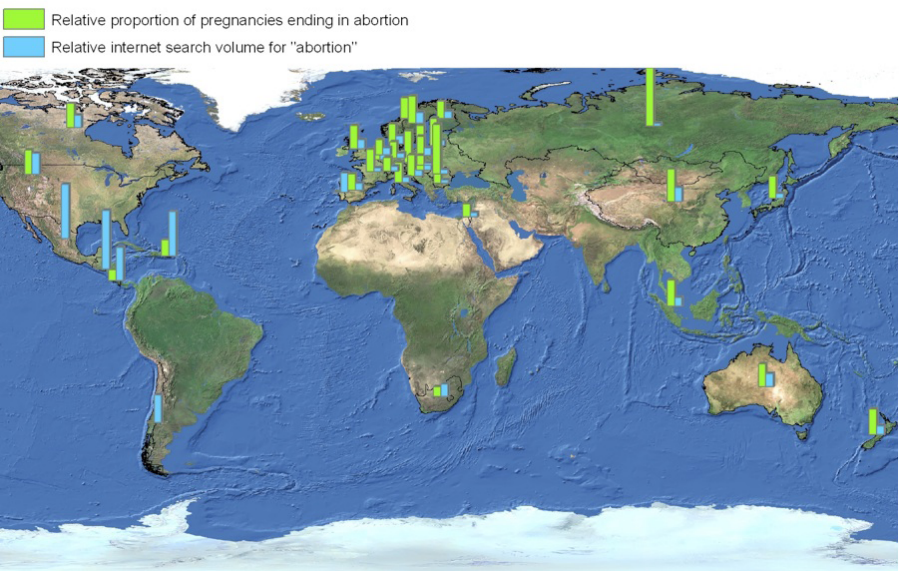
**Geographic visualization of abortion search volume and abortion rates across 37 countries**.

In the international analysis of 37 countries, search volume for abortion was also inversely proportional to local abortion rates (Rho = -0.484, p = 0.004; Figures 3, 4. Low abortion rates and high search volumes were seen in many Central and South American nations. High abortion rates and low abortion search volumes were seen in most Eastern European nations, with the sole exception of Poland, which had low abortion rates and low search volumes. These differences may be the result of differences in underlying attitudes towards abortion, which may be associated with cultural norms and/or with the pervasiveness and depth of religious adherence in these countries. Such potential associations would need to be studied in greater detail before any conclusions about them could be drawn.

## Discussion

Our case study examining the relationship between abortion policies and abortion rates reveals the potential value of Internet search patterns as a complementary measure of the impact of health policies. Perhaps the most interesting overall finding from this initial analysis is that abortion search volumes had a significant *inverse *relationship to local abortion rates and availability, and a significant *direct *relationship to the legislative and policy restrictions on abortion available for study. One likely interpretation of these findings is that individuals with limited access to local abortion services typically use the Internet to search for providers outside their area, while those with greater access to local abortion services may access them through local channels and are thus less likely to turn to the Internet to find an abortion provider. This interpretation is consistent with published evidence that local restrictions lead individuals to seek abortion services outside their area [11,12].

Another interesting finding is that the coastal US states (blue, Figure 1) tended to have higher abortion rates and lower abortion search volumes than non-coastal states (red). Differences in abortion rates and abortion search volumes were also observed between different regions of the world (Figure 3), as mentioned above. These differences are likely due to general historical, cultural and societal differences between the various regions. These general trends and their outliers require further investigation before conclusions can be drawn about causal relationships.

Further validation of these Internet search data has the potential to provide a valuable complementary data source for measuring the effects of health policies. Basic privacy issues around search data make accessing individual search records for the purposes of validation unfeasible. Furthermore, Internet-based surveys of this topic would be difficult given the sensitivity of the topic. Therefore, a fuller validation of these methods would require a resource intensive observational study. Such a study could involve classic community-based survey or potentially a survey performed at the clinics themselves to determine what the individuals were seeking in their Internet searches, why they chose to search on the Internet as opposed to other means, whether they eventually received an abortion, and if so, where the abortion was performed. Such a study could then be used to further examine the relationships between abortion-related searches, individual searchers' motivations, and rates of procedures performed. This validation could only take place in certain settings, as many of the countries where search results are available would not allow such an analysis for a variety of political or other reasons, highlighting one of the potential advantages of such data to overcome international barriers to health policy research.

Search data have a number of advantages over traditional surveillance sources that make them attractive as potential measures of health outcomes and policies: *Timeliness *- while traditional data often take years to collect, search data are available in near-real-time; *Efficiency *- while traditional measures require dedicated reporting infrastructure in each region, and many countries do not have strong public health reporting infrastructures, search data may be automatically collected in a centralized fashion; and *Transparency *- as mentioned above, search data are available outside traditional reporting channels and thus subject to less government censorship and control.

Limitations of search data include varying online access and Internet search usage across different demographic, socioeconomic and geographic subpopulations, and searches for abortion by individuals who are not seeking abortion services. Evaluation across a range of different settings and topics will provide a deeper understanding of the strengths and weaknesses of these data. Evaluation will also further clarify the potential benefits of conducting analyses based on data collected over long periods of time, compared to data collected over shorter periods of time that may be subject to a variety of transient effects. Furthermore, access to information on illegal abortions, may provide additional insight into the value of Internet query surveillance.

## Conclusions

Due to their significant advantages in timeliness, efficiency, and transparency, search data have the potential, with adequate validation, to become an important complementary data source for researchers and decision-makers studying the effects of public health policies in local and international settings.

## Competing interests

The authors declare that they have no competing interests.

## Authors' contributions

BR and JB conceived of the study, collected and analyzed data and wrote and approved the final manuscript.

## Pre-publication history

The pre-publication history for this paper can be accessed here:

http://www.biomedcentral.com/1471-2458/10/514/prepub
